# Increased peripheral and local soluble FGL2 in the recovery of renal ischemia reperfusion injury in a porcine kidney auto-transplantation model

**DOI:** 10.1186/1479-5876-12-53

**Published:** 2014-02-23

**Authors:** Zitong Zhao, Cheng Yang, Long Li, Tian Zhao, Lingyan Wang, Ruiming Rong, Bin Yang, Ming Xu, Tongyu Zhu

**Affiliations:** 1 Department of Urology, Zhongshan Hospital, Fudan University; Shanghai Key Laboratory of Organ Transplantation, 180 Fenglin Road, Shanghai 200032, China; 2 Shanghai Key Laboratory of Organ Transplantation, 180 Fenglin Road, Shanghai 200032, China; 3 Transplant Group, Department of Infection, Immunity and Inflammation, University of Leicester, Leicester General Hospital, University Hospitals of Leicester, Leicester LE5 4PW, UK; 4 Biomedical Research Center, Zhongshan Hospital, Fudan University, Shanghai 200032, China; 5 Department of Transfusion, Zhongshan Hospital, Fudan University, Shanghai, China; 6 Qingpu Branch Zhongshan Hospital, Fudan University, 1158 Gongyuan Road East, Shanghai 201700, China

**Keywords:** Soluble FGL2, Ischemia reperfusion injury, Kidney auto-transplantation, Porcine, FcγRIIB

## Abstract

**Background:**

Regulatory T cells (Treg) protect kidney against ischemia reperfusion (IR) injury via suppressing innate immunity, but the mechanism has not been fully clarified. Soluble fibrinogen-like protein 2 (sFGL2), a novel effector of Treg, may affect apoptosis and inflammation. This study investigated the role of sFGL2 in renal IR injury in a porcine kidney auto-transplantation model.

**Materials and methods:**

The left kidney was retrieved from mini pigs and infused by University of Wisconsin solution into the renal artery with the renal artery and vein clamped for 24-h cold storage. After the right nephrectomy, the left kidney was auto-transplanted into the right for 2 weeks. 3 pigs were sacrificed at day 2, 5, 7, 10 and 14 post-transplantation respectively. Collected renal tissues and daily blood samples were stored for further analyses.

**Results:**

Both serum creatinine and blood urea nitrogen were maximized during day 2 to 5 and followed by a gradual recovery over 2 weeks. The similar pattern were showed in histological damage, myeloperoxidase + cells and apoptosis in the kidney, as well as circulating TNF-α and IFN-γ. Serum sFGL2 presented a fluctuating increase and reached a peak at day 10. The expression of sFGL2 and its receptor FcγRIIB as well as Foxp3 and IL-10 in the kidney was notably increased from day 5 to 10.

**Conclusion:**

The increased sFGL2 together with FcγRIIB during renal recovery after IR injury suggested that sFGL2 might be a potential renoprotective mediator involved in the renal self-repairing and remodeling in this 2-week porcine auto-transplantation model.

## Introduction

Kidney transplantation serves as the best treatment for end-stage renal failure patients, although many problems related with graft and patient survival have not been solved yet. Ischemia reperfusion (IR) injury, an inevitable issue in transplantation, contributes to delayed graft function, acute and chronic rejection [1,2]. IR injury induced inflammation and apoptosis involve in both the innate and adaptive immunity, and jeopardize allograft function eventually. Regulatory T cells (Treg) were shown to protect kidney from IR injury through its immunosuppressive properties in the innate immunity [3], whereas its mechanism has not been fully clarified.

Soluble fibrinogen-like protein 2 (sFGL2) is identified as a member of fibrinogen-related protein superfamily [4]. There are two different forms of FGL2 exerting distinct properties. One is FGL2 prothrombinase, the membrane-protein form, mainly expressed on the reticuloendothelial cells and exerts procoagulative activity [5]. The other is sFGL2, the soluble form, constitutively expressed by CD4^+^ and CD8^+^ T cells, while Treg cells inducibly secrete it [4]. sFGL2 works as a novel effector of Treg, demonstrating immunoregulatory function to protect against tissue injuries [6]. sFGL2 inhibited dendritic cell maturation and induced B cell apoptosis *in vitro*, while the downregulation of sFGL2 improved T cell proliferation, promoted Th1 cell polarization and inhibited Treg activity [7]. However, sFGL2 was recently shown contradictory properties as it promoted cellular apoptosis, such as sinusoidal endothelial cells and hepatocytes through binding to its inhibitory FcγRIIB receptor on the cell surface and led to tissue injuries [8].

In the previous study, we found that serum sFGL2 increased among renal allograft recipients with acute rejection (AR) to an extent dependent on pathological severity [9], and its secretion was induced by pro-inflammatory cytokines such as tumor necrosis factor (TNF) -α and interferon (IFN) -γ through MAPK pathway [10]. We also discovered that sFGL2, correlated with circulating Treg in AR patients, induced tubular epithelial cell (TEC) apoptosis [11]. Apoptosis is a process of programmed cell death serving as a defense mechanism to remove unwanted and potentially dangerous cells [12]. TEC apoptosis is a double-edged sword exerting contradictory effects in distinct stages of tissue injuries, destructive in the early and protective in the late [13]. sFGL2 induces apoptosis in both inflammatory cells and TECs. Since apoptosis of different type of cells could lead to different outcomes, the final impact of sFGL2 on the kidney varies in a spatial temper manner. However, current studies of sFGL2 in the kidney are limited to its level in circulation without continuous assessments. Therefore, this study aims to further investigate local expression of sFGL2 in injured kidneys and its change trend throughout the duration of 2 weeks in a porcine kidney auto-transplantation model.

## Materials and methods

### Animals

Under the regulation laid down by the Chinese animal welfare authority and the guidelines of the Care and Use of Laboratory Animals of the Laboratory Animal Ethical Commission of Zhongshan Hospital, Fudan University, 15 male mini pigs (Bama, Guangxi, China) weighing 25-30 kg and between 7-8 months of age were used. They were housed with air condition, straw saw dust beds, and free access to water and fed with wetted granulated full fodder.

### Anesthetic protocol

The animals were premedicated with 0.5 mg/kg of diazepam and 5 mg/kg of ketamine hydrochloride intramuscularly, followed by general anesthesia using 1 mg/kg of propofol (Fresenius Kabi, Bad Homburg, Germany) intravenously (i.v.), and maintained with a mix solution of 0.25 mg/kg/h of diazepam, 2.5 mg/kg of ketamine hydrochloride, and 0.0125 mL/kg/h of compound detomidine hydrochloride or 0.5 mg/kg/h of propofol i.v. in turn. The respiration was supported by a ventilator (Dräger, Lübeck, Germany) through an inserted trachea cannula. Five hundred milliliters of 5% glucose and 0.9% sodium chloride and 500 mL of hydroxyethyl starch 130/0.4 and sodium chloride injection (Fresenius Kabi, Bad Homburg, Germany) were also administered i.v.. In addition, 100 mL of 0.3 g of levofloxacin lactate and 2 million units of benzylpenicillin were given i.v. 30 min before surgery. The same anesthetic protocol was used for donor retrieving and transplantation.

### Donor kidney retrieving and preservation

The left kidney was mobilized and removed with minimal warm ischemia (about 1 min) after ligating the renal artery near the abdominal aorta, renal vein near the inferior vena cava, and ureter. The isolated kidney was flushed immediately with 200 mL of precooled Ringer solution with 1000 IU of heparin at 100 cm H_2_O hydrostatic pressure until the kidney became pale and then followed by 200 mL of the University of Wisconsin (UW; Bristol-Myers Squibb, New York, USA) solution. The kidney was preserved in the Steri-Drape (3 M Health Care, St. Paul, USA) with the iced UW solution at 4°C for 24 h.

### Right nephrectomy and auto-transplantation

Next day, the right kidney was resected after ligating the right renal artery and vein, as well as the ureter, close to the renal hilum. The left kidney was orthotopically auto-transplanted into the right. In addition, a double lumen cuffed silicone vascular access catheter (Arrow International, Reading, PA, USA) was placed in the left internal jugular vein. The lumens of the central line were fixed behind the ear and blocked with heparin.

During the period of 2 weeks, blood samples were taken daily. Three pigs were anaesthetized and sacrificed for the graft harvest at day 2, 5, 7, 10 and 14 post transplantation respectively. The right post-nephrectomy kidney was used as the normal control. The part of renal tissues was fixed with 10% buffered formalin for histological examination and the others were snapping frozen for molecular biological analyses.

### Histological assessment

The renal histological damage was assessed in Hematoxylin and Eosin (H&E) stained sections by three researchers blinded to the coding. The semi-quantitative score system was comprised of four criteria including loss of tubular epithelium, tubular vacuolation and nuclear loss, protein casts and interstitial expansion caused by oedema and/or cellular infiltration. Each criterion was graded from mild to severe by the percentage of injury: 0 (<1%); 1 (1–25%); 2 (26–50%); 3 (51–75%) and 4 (>75%).

### The serum level of sFGL2, TNF-α and IFN-γ

Peripheral blood samples were centrifuged at 4°C, 2500 rpm, for 25 min to obtain the serum. The serum level of sFGL2 was detected in duplicate using an enzyme-linked immunosorbent assay (ELISA) kit (Biolegend, San Diego, USA). The serum level of TNF-α and IFN-γ were also assessed in duplicate using ELISA kits (BD Biosciences, San Diego, USA). All experimental procedures were performed according to the manufacturer's instructions. The final concentration was determined using a standard curve.

### *In situ* end-labelling (ISEL) apoptotic cells

Paraffin sections were used for ISEL fragmented DNAs with digoxigenin-deoxyuridine (dUTP) by terminal deoxynucleotidyl transferase (TdT) using an Apoptosis Detection Kit (Millipore, Billerica, USA). Briefly, the sections were digested by 40 μg/ml proteinase K for 15 min at 37°C, incubated with TdT and digoxigenin-dUTP at 37°C for 60 min and transferred to wash/stop buffer for 30 min. After adding anti-digoxigenin-peroxidase complex for 30 min, these sections were developed by AEC substrate. Apoptotic cells were examined at 400× magnification for 20 fields in tubulointerstitial areas.

### Immunostaining for myeloperoxidase (MPO), active caspase-3, sFGL2 and FcγRIIB

For antigen retrieval, paraffin sections were digested by the same method with ISEL for MPO, or using 10 mM sodium citrate buffer for active caspase-3, sFGL2 and FcγRIIB. The sections were then blocked by peroxidase-blocking reagent and labelled by anti-MPO (1:600 dilution, DAKO), anti-active caspase-3 antibody (1:100 dilution, R&D System, Minnesota, USA), anti-sFGL2 (1:200 dilution, Abcam, Cambridge, UK) or anti-FcγRIIB (1:200 dilution, Abcam) antibodies at 4°C overnight. The antibody binding was revealed by AEC for MPO and active caspase-3, and DAB for sFGL2 and FcγRIIB. MPO + cells and active caspase-3+ cells in the renal cortex were manually counted, while the expression of sFGL2 and FcγRIIB were semi-quantitatively scored using optical volume density (OD × mm^2^) analysis (Image-Pro Plus 6.0, Media Cybernetics Inc., Bethesda, USA) in 20 fields at 400× magnification.

### Protein expression assay by western blotting

Twenty μg protein from kidney homogenate was separated on 15% (wt/vol) poly acrylamide denaturing gels and electro-blotted onto Hybond-C membranes. These membranes were blocked with 5% (wt/vol) milk, separately probed with anti-active caspase-3 (1:1,000 dilution, Cell Signaling Technology, Boston, USA), anti-soluble FGL2 (1:10,000 dilution, Abcam) or anti-FcγRIIB (1:10,000 dilution, LifeSpan BioSciences, Seattle, USA) antibody. For the loading control, the same membranes were probed with anti-β-actin antibody (1:10,000 dilution, Abcam), and then incubated with peroxidase-conjugated secondary antibodies (1:10,000 dilution, Jackson ImmunoResearch, West Grove, USA) at room temperature for 1 h. Immunoreactive bands were visualized using ECL substrate (Thermo Fisher Scientific, Rockford, USA) and a Bio-Image Analysis System (Cell Biosciences, Inc., Santa Clara, USA). The semi-quantitative analysis results were expressed as optical volume density (OD × mm^2^) and normalized by β-actin for loading (AlphaView Software 3.3, Cell Biosciences, Inc.).

### Foxp3 and IL-10 mRNA expression

Total RNA was extracted from renal tissues with Trizol reagent (Invitrogen, Carlsbad, USA). One μg of total RNA was transcribed into cDNA using a RevertAid™ First Strand cDNA Synthesis Kit (Fermentas, Glen Burnie, USA). Real-time quantitative PCR (qPCR) was performed using the SYBR Premix Ex Taq Kit (Takara Bio Inc., Otsu, Japan) in a MasterCycler RealPlex4 system (Eppendorf, Hamburg, Germany). After a hot start (30 seconds at 95°C), amplification was performed for 45 cycles (5 seconds at 95°C, 30 seconds at 55°C, 60 seconds at 72°C). The primers were listed in Table 1. The expression of mRNA normalized with GAPDH were calculated against control kidneys (Post N) using a 2^-ΔΔCt^ method.

**Table 1:** The sequences of the primers

**Gene name**		**Primer sequence**
GAPDH	Sense	TTCCACGGCACAGTCAA
	Antisense	GCAGGTCAGGTCCACAA
IL-10	Sense	AGCCAGCATTAAGTCTGAGAA
	Antisense	CCTCTCTTGGAGCTTGCTAA
Foxp3	Sense	CTCCTACTCCCTGCTGGCAAAT
	Antisense	CGCATGTTGTGGAACTTGAAGTAGT

### Statistical analysis

Results are expressed as mean ± standard error of the mean (SEM). Normality tests were carried out. One-way ANOVA was used for statistics, and post hoc comparisons were then performed by Scheffe test using SPSS 18.0 software (SPSS Inc, Armonk, USA). P values <0.05 were considered to be statistically significant.

## Results

### Renal function and histological damage

**Figure 1:**
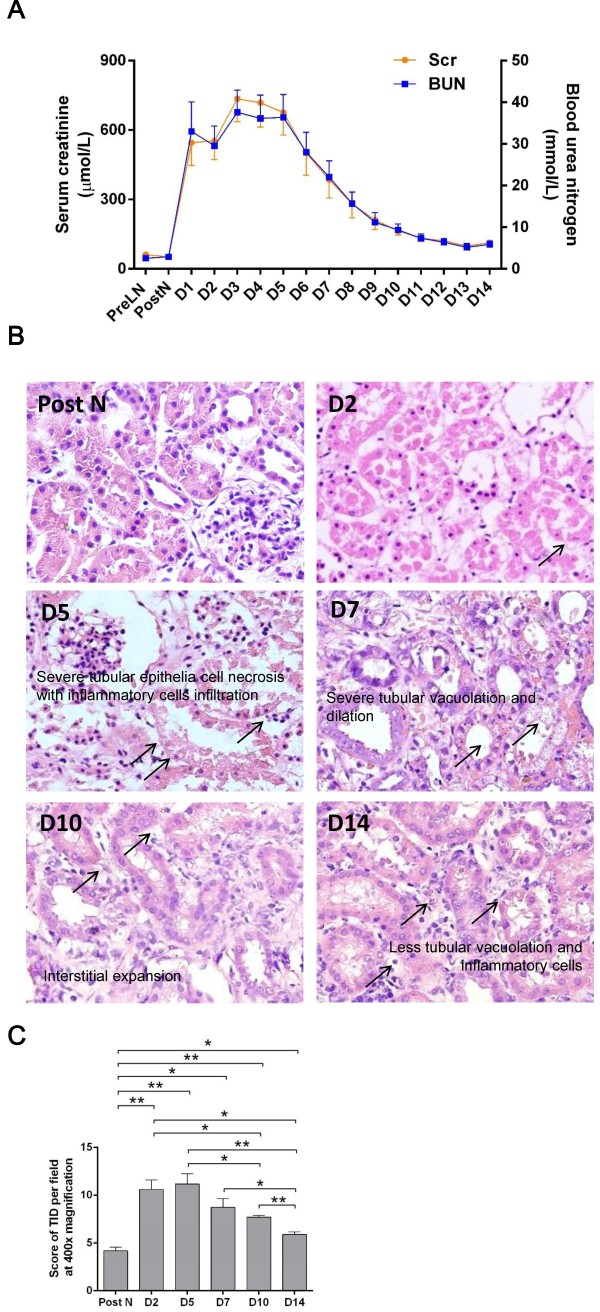
**Renal function and renal tissue damage.** Serum creatinine and urea nitrogen sharply increased at day 1, reached a plateau at day 3 to 5 and fell close to normal at day 14 **(A)**. Tubular vacuolation and detachment, as well as protein casts, interstitial expansion and cellular infiltration were seen in the posttranplant kidneys **(B)**. The score of tubulointerstitial damage (TID) assessed in H&E sections at 400× magnification showed that renal tissue damage was significantly more severe at day 2 and 5 post transplantation than post nephrectomy, marginally better at day 7, followed by gradual recovery at day 10 and 14 **(C)**. Data are expressed as mean ± SEM (n = 3). Pre LN: pre left nephrectomy; Post N: post nephrectomy. *: *P* < 0.05; **: *P* < 0.01.

Serum creatinine (Scr) and blood urea nitrogen (BUN) were examined before left nephrectomy and daily over 14 days after auto-transplantation. The level of both Scr and BUN sharply increased at day 1, reached a plateau at day 3 to 5, fell off gradually thereafter and closed to the normal level at day 14 (Figure 1A).

The tubulointerstitial damage (TID) was assessed in H&E stained sections of the grafts harvested at day 2, 5, 7, 10 and 14 post-transplantation. Compared with the normal tissue from right nephrectomy, there were lots of tubular vacuolation and detachment, as well as protein casts, interstitial expansion and cellular infiltration in the post-tranplant kidneys at day 2 and 5. TID became less severe at day 7 and 10, but mild tubular dilation and interstitial expansion were still seen at day 14 (Figure 1B). The semi-quantitative analysis revealed that TID was significantly more severe at day 2 and 5 post-transplantation than normal tissue from post-nephrectomy, marginally better at day 7, followed by a gradual recovery from day 10 to 14 (Figure 1C).

### Inflammation in the circulation and kidney

**Figure 2:**
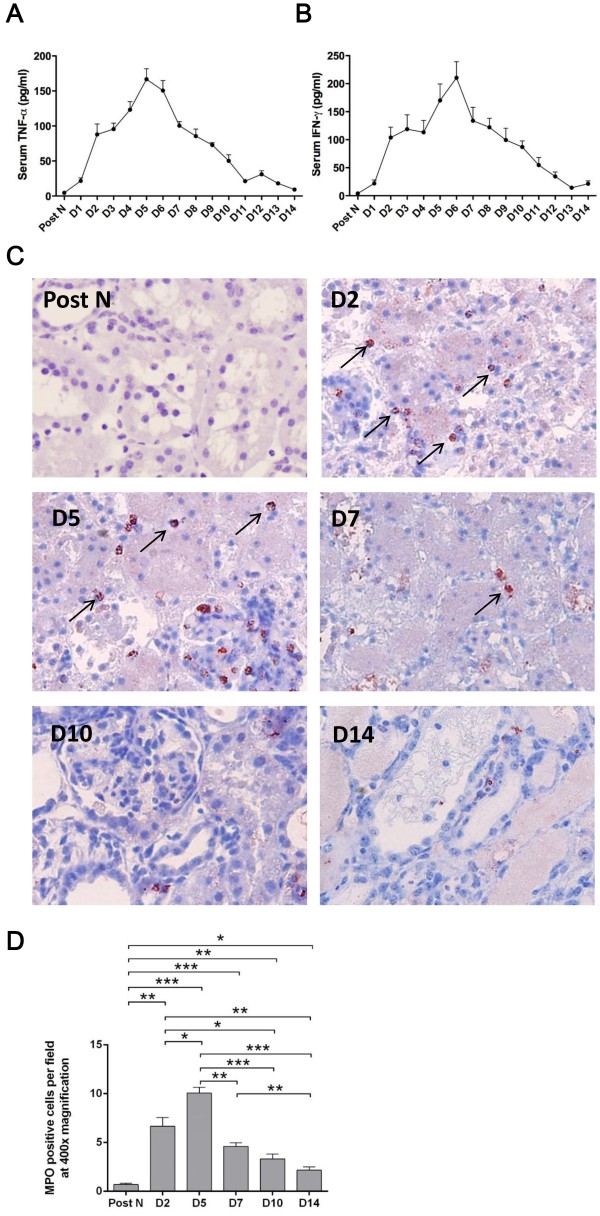
**Inflammation in the peripheral blood and kidney.** The serum level of TNF-α **(A)** and IFN-γ **(B)** showed a stable increase after transplantation, a summit at day 5 and a gradual decrease thereafter. Immunohistochemistry staining at 400× magnification displayed that MPO + cells scattered in the posttransplant kidneys, mostly in vascular lumens and interstitial areas and some also in glomerular areas, or penetrating through tubular areas **(C)**. The number of MPO + cells markedly increased from day 2, culminated at day 5 and notably reduced since day 7 till day 14 **(D)**. Data are expressed as mean ± SEM (n = 3). Post N: post nephrectomy. *: *P* < 0.05; **: *P* < 0.01; ***: *P* < 0.001.

To investigate the profile of systemic inflammatory responses *in vivo* during the processes of injury and recovery, the serum level of pro-inflammatory cytokines TNF-α and IFN-γ, as well as neutrophils infiltration in the kidney, was detected. The similar trends were shown as renal function with continuous increase post transplantation, a summit at day 5 or day 6 and a gradual decrease thenceforth (Figure 2A-B).

The MPO + cells in the kidney were scattered in the post-transplant kidneys, mostly in vascular lumens and interstitial areas and some also in glomerular areas, or penetrating through tubular areas (Figure 2C). The number of MPO + cells was markedly increased from day 2, culminated at day 5 and notably reduced since day 7 till day 14 (Figure 2D).

### Apoptotic cells in the kidney

**Figure 3:**
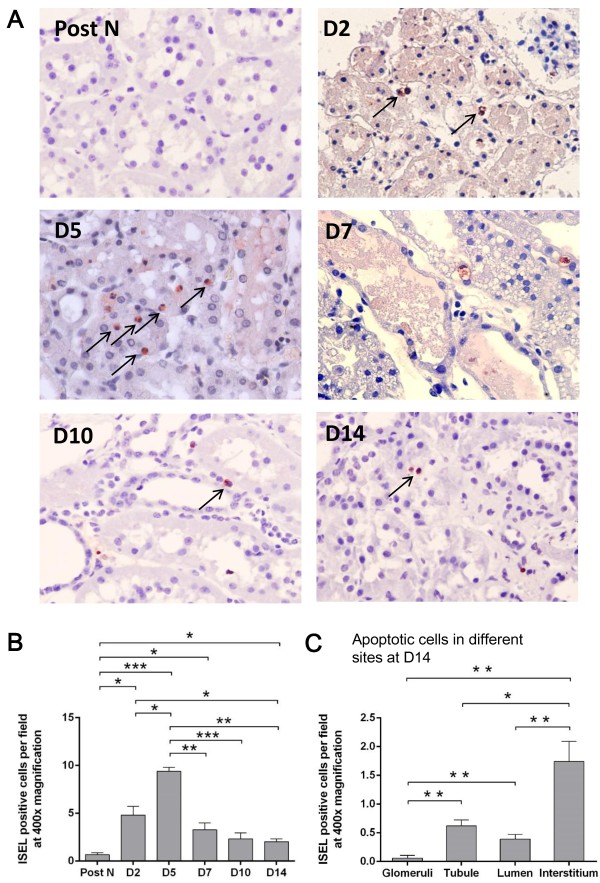
**ISEL + cells in the kidney.** Detected by ISEL fragmented DNAs at 400× magnification, apoptotic cells were hardly seen in the post-nephrectomy kidneys, but increased after transplantation, which were mainly located in tubulointerstitial areas and some were shedding into tubular lumens **(A)**. The number of apoptotic cells was remarkably higher at day 2 than post nephrectomy, peaked at day 5 and significantly decreased thenceforth **(B)**. At day 14 post transplantation, most apoptotic cells were observed in the interstitial area **(C)**. Data are expressed as mean ± SEM (n = 3). Post N: post nephrectomy. *: *P* < 0.05; **: *P* < 0.01; ***: *P* < 0.001.

**Figure 4:**
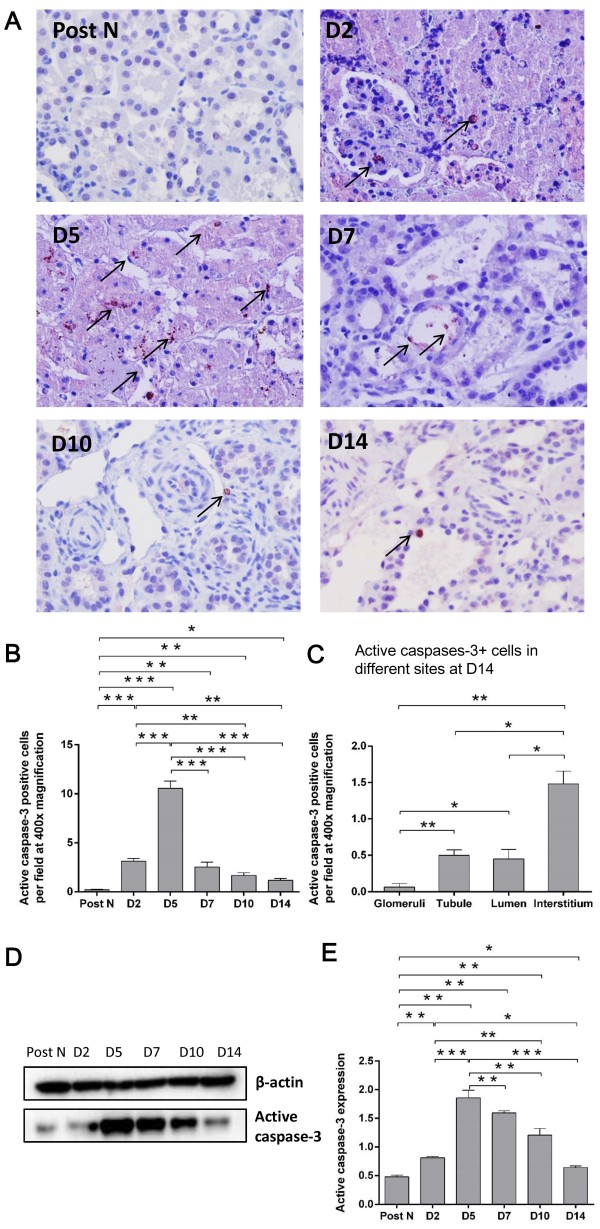
**Active caspase-3 expression in the kidney.** Active caspase-3+ apoptotic cells were increased post-transplantation, mainly located in the tubule and tubulointerstitial area with some shedding into tubular lumens **(A)**. The number of active caspases-3+ cells was significantly higher at day 2 than post nephrectomy, peaked at day 5 and remarkably decreased thenceforth **(B)**. Similar to ISEL + cells, most active caspase-3+ cells were observed in the interstitial area at day 14 post transplantation **(C)**. Accordingly, the protein expression of active caspase-3 was consistent with its immunostaining results **(D-E)**. Data are expressed as mean ± SEM (n = 3). Post N: post nephrectomy. *: *P* < 0.05; **: *P* < 0.01; ***: *P* < 0.001.

Apoptosis also represents the degree of renal allograft injury after transplantation. Therefore, the number of apoptotic cells was detected in the kidney using ISEL and active caspase-3 labeling. Apoptotic cells were hardly seen in the post-nephrectomy kidneys, but increased after transplantation, which were mainly located in tubulointerstitial areas and some were shedding into tubular lumens (Figures 3A and 4A). The dynamic change in the number of apoptotic cells within 2 weeks resembled that of MPO + cells, which was remarkably higher at day 2 than post nephrectomy, peaked at day 5 and significantly decreased thereafter (Figures 3B and 4B). It was interesting that most apoptotic cells were observed in the interstitial area rather than the glomerular, tubular or lumen areas at day 14 after transplantation, when the renal structural reconstruction and recovery were dominant (Figures 3C and 4C). The protein expression of active caspase-3 was in accordance with its immunostaining results, except that relative higher levels were seen from day 7 to 14, which might be due to higher sensitivity of western blotting compared to immunostaining (Figure 4D-E).

### sFGL2 expression in the circulation and kidney

**Figure 5:**
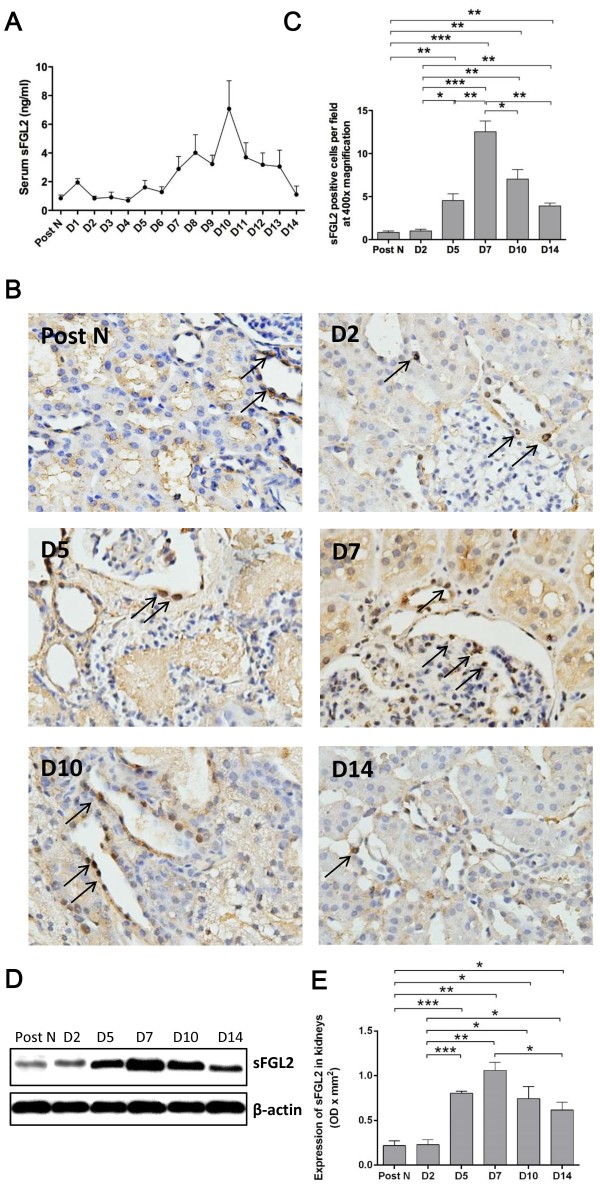
**The peripheral and local expression of sFGL2.** Serum sFGL2 presented a fluctuating increase after transplantation, reached the peak at day 10 and gradually fell off eversince **(A)**. Immunohistochemistry staining at 400× magnification showed that sFGL2 positive cells included TECs, interstitial cells and a few in the glomeruli **(B)**. The number of sFGL2 positive cells in the kidney **(C)** and western blotting **(D-E)** revealed that there was no significant difference in the expression of sFGL2 between normal renal tissue and that of day 2 post transplantation. sFGL2 in the kidney remarkably increased from day 5, culminated at day 7 and gradually decreased at day 10 and 14. Data are expressed as mean ± SEM (n = 3). Post N: post nephrectomy. *: *P* < 0.05; **: *P* < 0.01; ***: *P* < 0.001.

The level of sFGL2 in the peripheral blood was detected by ELISA. Serum sFGL2 presented a fluctuating increase after transplantation, reached the peak at day 10 and gradually decreased thereafter (Figure 5A). The localization and expression of sFGL2 in the kidney were determined by immunohistochemistry staining and western blotting. Different kinds of sFGL2 positive cells by immunohistochemistry staining were observed including TECs and other cells in the interstitial or glomeruli area, which represented the secreting cells or the target cells of sFGL2 (Figure 5B). There was no significant difference in the expression of sFGL2 between the normal tissues and that of day 2 post-transplantation. sFGL2 in the kidney remarkably increased from day 5, culminated at day 7 and gradually decreased at day 10 and 14 (Figure 5C-E).

### FcγRIIB expression in the kidney

**Figure 6:**
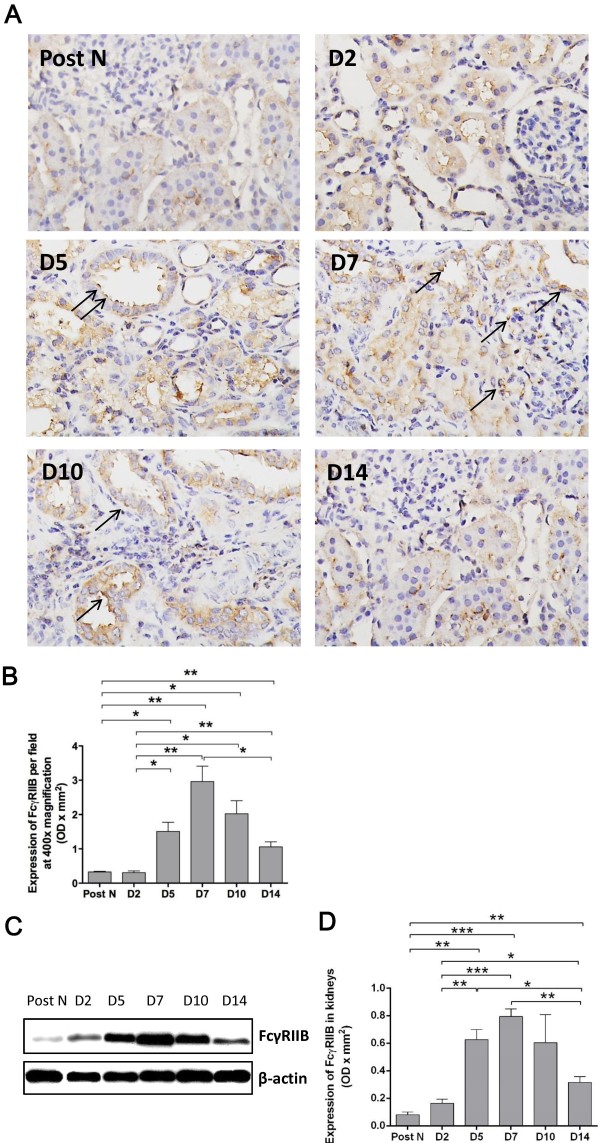
**FcγRIIB expression in the kidney.** Immunohistochemistry staining at 400× magnification revealed that FcγRIIB, the receptor of sFGL2, focused on TECs **(A)**. The number of FcγRIIB positive cells in the kidney **(B)** and western blotting **(C-D)** showed that after a slight shift at day 2, FcγRIIB significantly rose from day 5, followed by a peak at day 7 and a subsequent decrease at day 10 and 14. Data are expressed as mean ± SEM (n = 3). Post N: post nephrectomy. *: *P* < 0.05; **: *P* < 0.01; ***: *P* < 0.001.

Since the significant change of sFGL2 in the kidney autograft was observed, the localization and quantity of FcγRIIB in the kidneys were subsequently examined. FcγRIIB, the receptor of sFGL2, was mainly displayed on TECs, showing the main target of sFGL2 (Figure 6A). The change trend of FcγRIIB during 2 weeks post-transplantation was analogous to that of sFGL2. After a slight low expression at day 2, FcγRIIB was significantly risen from day 5, followed by a peak at day 7 and a subsequent decrease at day 10 and 14 (Figure 6B-D). The synchronical change of sFGL2 and its receptor FcγRIIB further testified the hypothesis that sFGL2 was involved in the recovery of renal IR injury.

### Foxp3 and IL-10 expression in the kidney

**Figure 7:**
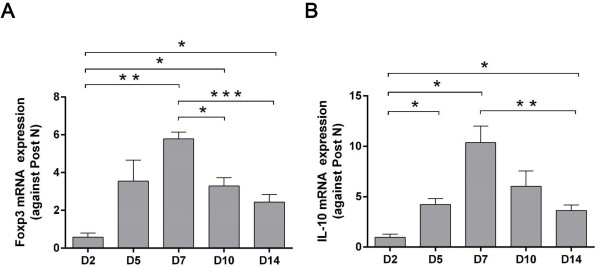
**Foxp3 and IL-10 mRNA expression in the kidney.** Both Foxp3 and IL-10 mRNA in the kidney remarkably increased from day 5, culminated at day 7 and gradually decreased at day 10 and 14 **(A-B)**. Data are expressed as mean ± SEM (n = 3). *: *P* < 0.05; **: *P* < 0.01; ***: *P* < 0.001.

Based on the dynamic changes of sFGL2 throughout 2 weeks of renal ischemia reperfusion injury, its secreting cells, Tregs, were further examined by the expression of representing Foxp3 and IL-10 in the kidney. Both Foxp3 and IL-10 mRNA in the kidney remarkably increased from day 5, culminated at day 7 and gradually decreased at day 10 and 14 (Figure 7A-B), with a trend similar to that of sFGL2 expression in the kidney.

## Discussion

Our serial *in vivo* and *in vitro* studies showed that circulating sFGL2 was increased in kidney injuries and contributed to TEC apoptosis [11]. Here, we further investigated the dynamic change of sFGL2 in the kidney and circulation throughout 2-week duration of renal IR injury in a porcine auto-transplantation model. This study revealed that renal dysfunction and histological damage was maximized from day 2 to 5 post-transplantation and followed by a gradual recovery, while the expression of sFGL2 and its receptor FcγRIIB exhibited a delayed peak at day 5 to 10. These results indicate that sFGL2 might be involved in the process of renal repairing and remodeling and exert protective effects.

sFGL2, as an immunoregulatory effector of Treg, has been proved a capacity of TEC apoptotic induction. TECs compromise over 80% of renal parenchymal cells and play an important role in maintaining normal kidney function [14]. TEC apoptosis is closely related with IR injury, delayed graft function and early allograft survival [15]. It was shown that TEC apoptosis occurs in two distinct phases of kidney injuries. The first one occurs early on 12 to 48 h after the injury, during which TEC apoptosis leads to tubular cell loss and tubular dysfunction. Whereas the second phase occurs days later in the recovery process, with TEC apoptosis postulated to contribute to the remodeling of injured tubules and to facilitate their return to a normal structural and functional state [13]. Although raised TNF-α and IFN-γ in the early renal IR injury could promote sFGL2 production, the delayed peak of sFGL2 at the recovery stage was likely to be further facilitated by other causes apart from these two pro-inflammatory factors. TEC apoptosis at the early stage of renal injury is possibly due to other factors but sFGL2.

Apoptosis is a complex process involving multiple factors, among which TNF-α is a typical apoptosis-inducing mediator [16]. There are two broad pathways leading to apoptosis, the extrinsic and intrinsic pathways [17]. The extrinsic apoptosis indicates a form of death induced by extracellular signals that result in the binding of ligands, such as TNF-α, to specific trans-membrane receptors, collectively known as death receptors belong to the TNF superfamily, and recruiting the down-stream adaptor proteins. The intrinsic apoptosis, on the other hand, is activated in response to a number of stressing conditions, converges on the mitochondria and leads to the release of cytochrome C. In both pathways, signaling results in the activation of a family of cysteine proteases, named caspases, which acts in a proteolytic cascade to dismantle and remove the dying cells. This study showed the strikingly increase of TNF-α together with the maximized TEC apoptosis, renal function and tissue damage at day 5, indicating a link and cascade events among them, as well as a contribution of TEC apoptosis to the loss of renal function.

Moreover, apoptotic cells, featured with nuclear and cytoplasmic condensation, followed by plasma membrane blebbing and releasing apoptotic bodies, are rapidly identified by neighboring cells or professional phagocytes and dispose generally without induction of inflammation [18]. The slight increase of sFGL2 at the peak of renal IR injury probably initiates renal recovery by inducing apoptosis in inflammatory cells in the interstitial area and limiting inflammation expansion. Taken together, the change trend of MPO + cells with a peak at day 5 and a subsequent decrease, consistent with that of circulating pro-inflammatory cytokines, was linked to the severity changes of renal injury in terms of renal function and tubulointerstitial damage throughout 2-week post auto-transplantation. Most porcine kidney auto-transplantation studies vary in modeling duration and renal recovery pattern. In a UW-preserved 7-day porcine kidney auto-transplantation [19], renal function continuously deteriorated till day 7, while its only histology assessment at day 7 might limited the knowledge of kidney responses in between. In another 10-week study with similar modeling to ours [20], the peak of renal IR injury was at 2 to 4 days as well followed by gradual renal function recovery. However, compared to its one-time intra-operative biopsy showing early reperfusion injury, our study exhibited a more comprehensive inflammatory and apoptosis trend in the kidney throughout 2-week duration. The wide variations among studies might be owing to different surgical techniques and perioperative care.

Most interestingly, the highest level of sFGL2 during a period of 2-week auto-transplantation appeared approximately 2 to 5 days later than that of renal function and histological damage, as well as inflammation and apoptosis. It is recently demonstrated that Treg modulate renal IR injury through IL-10-mediated blockade of innate immune cell activation and accumulation [6]. The data from this study indicate that the enhanced injury might trigger the process of repairing and remodeling, which was marked by the increased expression of sFGL2. The consistent changes of sFGL2 and its receptor FcγRIIB with Foxp3 and IL-10, representative of Treg cells, throughout 2 weeks reveal that our study provides sFGL2 as another potential mechanism of Treg protecting renal IR injury. Undoubtedly, further intervention researches are needed to further support our hypothesis. Moreover, the expression of sFGL2 in the kidney peaked at day 7 while its serum level at day 10, probably owing to the initial great transfer of circulating sFGL2 into the kidney for recovery but less convey after its renoprotection fulfilled. Apparently, activated Treg recruited to the injured kidney and secreting large amounts of sFGL2 locally at the beginning of renal recovery is also a likely explanation.

In conclusion, our findings provide a new insight into renal IR injury throughout 2-week duration in a porcine kidney auto-transplantation model. Taken together with the 2-week inflammatory and apoptosis trend in renal ischemia reperfusion injury, the continuous assessment of sFGL2 expression in the peripheral blood and renal tissues reveals the potential involvement of sFGL2 in renoprotection as a novel mechanism. However, the direct sFGL2-dependent effect of Treg in attenuating renal injuries needs to be further investigated.

## Competing interests

The authors have declared no competing interests.

## Authors’ contributions

ZZ and YC conceived of the study, and participated in the design of the study and performed the statistical analysis. YC, LL, ZT and XM carried out the animal surgery. LL and WL performed the pathological and molecular studies. ZZ and YC drafted the manuscript. YB participated in the design of the study and revised the manuscript. RR participated in the statistical analysis. XM and ZT provided the fund support. All authors read and approved the final manuscript.
